# Supplementary material for: Wildfire Smoke Exposure During Pregnancy: Consensus-Building to Co-Create a Community-Engaged Study
Source: Int J Environ Res Public Health. 2024 Nov 14;21(11):1513. doi: 10.3390/ijerph21111513 (PMC11593486; doi:10.3390/ijerph21111513)
Supplement: Supplementary file 1 [file ijerph-21-01513-s001.zip › ijerph-3279551-supplementary.pdf]

Supplemental Table S1. Level of Involvement, Expertise, and Interests Survey

**Question**	**Response Options**
**BLOCK 1**: Introductory text block and resources	
1. Thank you for your continued interest in the Wildfire and Infant Health Study. We have received funding to build a team that will plan a longitudinal study to assess impacts of prenatal exposure to wildfire smoke on infant health.<br><br>The next few questions are designed to understand your expertise and interest in the project, as well as to plan the next series of meetings.<br><br>Please fill out this survey even if you missed the November 9, 2021 Kick-off meeting.	n/a
2. Were you able to attend the November 9, 2021 Kick-off meeting?	Yes<br>No
3. f you missed the meeting, you can find meeting notes and a link to the recording here. It will be updated shortly after each meeting (1-2 days).	n/a
**Block 2:** Level of Involvement	
4. At the November 9, 2021 meeting, we discussed the goals of this team-building project. We are asking people to agree to the following:<br><br>• Attend online meetings* through March 2022 (8 meetings, around 1.5 hours each)<br>• Answer short (15-20 minute) surveys (n=5) to assist in planning meetings and designing the study<br>• Assist with content for a grant application (review and/or participate in writing)<br>• Connect with additional potential partners<br>• Identify existing data sources<br><br>*meeting dates/times will be determined by a poll in this survey and will be scheduled over the next week.	n/a
5. After reviewing the meeting materials and notes, what is the level of involvement you are comfortable with?	Multiple Choice (select one):<br><br>Team member - I would like to attend all meetings and help plan this study<br><br>Advisor - I will attend most meetings and provide input, but cannot be part of the research team<br><br>Observer - I would like to be aware of what the team is doing, but will

	not participate in planning<br><br>I am not sure at this point in time. I would like to learn more.<br><br>Unfortunately, I cannot be involved in this effort at this time.
**BLOCK 3**: Name Contact, Expertise	
6. Please enter your name (First Name, Last Name)	Open-text response
7. What is your email address?	Open-text response
8. Please describe your title and affiliation(s) that are most pertinent to this project. For example, "I am a community liaison concerned about wildfire smoke in Klamath County" or "I am an environmental epidemiologist."	Open-text response
9. Which of the following (you may select more than one) most closely aligns with your level of expertise or knowledge?<br>• Toxicology<br>• Epidemiology<br>• Maternal/infant health<br>• Air quality<br>• Chemistry<br>• Public health<br>• Clinical medicine<br>• Community engagement	Multiple choice (may select more than one)
10. If the above options do not reflect your area of knowledge, please describe here. Otherwise, you may skip this question.	Open-text response
11. Are you currently working on any projects related to wildfires or infant health? Please describe here if you can.	Open-text response
12. At our next meeting (see below for scheduling options) we will be discussing the basic elements of study design. Using the sliding scales below, please indicate your level of interest in helping design these components of the proposed study.<br>• Developing the research question & hypothesis<br>• Methods of community engagement (recruitment, return of data)<br>• Selecting the target population (geographic area, eligibility criteria)<br>• Selecting the health outcomes of interest<br>• Selecting chemicals/pollutants of concern<br>• Study methodology<br>• Selecting data collection tools (instrumentation, surveys, biological samples, etc.)<br>• Analyzing existing data sources<br>• Writing grant proposals	Slider<br>0 = no interest, 5 = high interest<br>Not sure<br>Other (open text response)

• Other (open text response)	
13. We have funding available to compensate individuals for their time on this project. If such funding would be helpful to support your involvement, please indicate that below.	Multiple choice (select one)<br><br>This is not necessary<br><br>I would like to learn more about funding options to support my involvement
14. We would like to determine availability for our next series of meetings. We anticipate meeting four more times before January 15. We understand you may not be able to attend all meetings, so will make recordings and notes available on our website.<br><br>Please mark your availability below. We are blocking off 2 hours, but will strive to keep meetings to 1.5 hours.	Available, Tentative, Not Available<br><br>Series of dates/times for each of the next 4 meetings.
15. Please use this space to provide any thoughts you may have about this project, your involvement, or other people you think we should reach out to.	Open-text response
16. If there are any papers or presentations you think we should be aware of, you may upload them here or email them to (contact email). For URLs, please enter them in the space above.	Upload documents/paste urls

Supplemental Table S2. Chemicals of Interest & Data Collection Tools Survey

**Question**	**Response Options**	**Embedded Information**
**BLOCK 1:** Introductory text		
1. Thank you for your continued interest! We are thrilled to see so many people coming together to learn more about the impact of wildfire smoke on infant health.<br><br>As a reminder, our meeting notes, recordings and other information are available on this webpage.	**n/a**	**n/a**
2. Over our next series of meetings, we are going to focus on specific study elements. The tentative meeting agenda is listed below. For this next meeting, we are going to focus on the chemicals of interest and potential data collection tools.<br><br>To make the best use of our time, we are using this survey to gather information from you regarding what type of information we should collect regarding wildfire smoke, and how we should collect that information.	**n/a**	**n/a**

<table>
<tr><td>Even if you are not familiar with some of these questions, we encourage you to look at the information provided.

Your answers to these questions help us learn more about your interests. We will continue to discuss all of this at the next meeting. We do not expect to make any decisions at this point, but will continue to use your input as we go through this process.

Meeting #2
Chemicals of interest
Data collection tools

Meeting #3
Health outcomes of interest
Timing of the study (time during pregnancy, before/during/after wildfires)

Meeting #4
Wrap-up discussions

Meeting #5
Discuss a small, Klamath-specific pilot project</td><td></td><td></td></tr>
<tr><td>3. At our first meeting, we briefly talked about different chemicals found in wildfire smoke. Most research has focused on PM2.5. We would like to hear from you how interested you are in looking at additional chemicals for this study. We have included links for you to learn more about these chemicals.

• Particulate Matter (PM2.5)
• Polycyclic aromatic hydrocarbons
• Flame Retardants
• Dioxins & furans
• Pesticides
• Endocrine disruptors
• Industrial products
• Personal care products
• Polychlorinated biphenyls
• Metals
• Other (open-text response)

Not sure what you might be interested in? You can mark that in the question below as well.</td><td>Slider
0 = no interest, 5 = high interest
Not sure.</td><td>Links to online resources embedded in topics listed in question.

$PM_{2.5}$:
www.epa.gov/pm-pollution/particulate-matter-pm-basics

PAHs:
https://ehsc.oregonstate.edu/resources/infographics?slide=polycyclic-aromatic-hydrocarbons

Flame retardants:
https://cehc2016annualreportdotcom.wordpress.com/wp-content/uploads/2017/01/flame-retardants-1.png</td></tr>
</table>

		**Dioxins and furans:** www.epa.gov/dioxin/learn-about-dioxin<br><br>**Pesticides:** http://npic.orst.edu/<br><br>**Endocrine disruptors:** www.epa.gov/endocrine-disruption<br><br>**Industrial chemicals**: www.industrialchemicals.gov.au/chemical-information/what-industrial-chemical<br><br>**Personal care products:** www.fda.gov/industry/fda-basics-industry/are-all-personal-care-products-regulated-cosmetics<br><br>**Polychlorinated biphenyls**: www.epa.gov/pcbs
4. We would also like to learn more about the types of data collection tools that would be a good fit for this study. These tools can range from surveys, to blood collection. There are many different types of ways to collect information. If you are not familiar with data collection tools, we have included some resources.<br><br>• This table shows some different tools and their associated pros and cons.<br>• We developed a short video about selecting different tools (you will need to scroll down to find it). It was developed for firefighters, but still provides a nice overview.	n/a	Links to table of tools and short video embedded.<br><br>**Table of tools:** https://www.fireengineering.com/health-safety/evaluating-fireground-exposures-why-the-sample-matters/<br><br>**Video**: https://fses.oregonstate.edu/chemical-exposures-first-responders
5. There are many different types of data that can be collected. In your mind, how important do you think each data type would be for this study?	Slider<br>0 = not important, 5 = very important<br>Not sure	Link to online resources embedded for Vital statistics: www.cdc.gov/nchs/about/factsheets/factshe

• Personal data collection<br>• Environmental data collection (sample air)<br>• Vital Statistics<br>• Surveys/ Questionnaires<br>• Health data (medical, records, lung function, etc)<br>• Other (open text response)<br><br>Not sure what you might be important? You can mark that in the question below as well.		et_nvss_improvements.htm
6. There are many tools to collect information about what a person is exposed to. This image shows several common ways to collect data from a person.	n/a	Common Personal Samplers graphic inserted.
7. In Meeting #2, we are focusing on the exposure side of the study. The next question will ask about the types of tools that can look at what a person, or a community, might be exposed to.<br><br>In Meeting #3 we will revisit this question as we discuss the health outcomes we are interested in. Ultimately we will want to choose data collection tools that are a good fit for capturing exposure, and for measuring health.<br><br>Therefore, for the next question, please think about tools from an exposure standpoint.	n/a	n/a
8. Using the sliding scales below, please indicate how interested you are in using these samplers as part of the study to capture exposure. In addition to the table above, we have included hyperlinked examples - we are not endorsing any products, just providing an example of what these tools can look like. For example, there are many personal PM2.5 monitors - we are showing just one example.<br><br>• Personal $PM_{2.5}$ monitor<br>• $PM_{2.5}$ monitor plus PUF for VOCs (see example in prior video)<br>• Silicone wristband<br>• Blood samples (umbilical blood, maternal blood)<br>• Placental sample<br>• Urine samples<br>• Other (open-text response)<br><br>Which are you most interested in?	Slider<br>0 = no interest, 5 = high interest	Links to online resources embedded for:<br>**Personal $PM_{2.5}$ monitor**:<br>www.amazon.com/Plume-Labs-Personal-Pollution-Sensor/dp/B07G9PTQW6<br>**Silicone wristband**:<br>https://youtu.be/Kmw7BCvcck8
9. As we prepare for future meetings, we would like to be concurrently thinking about health outcomes of interest. The following health outcomes have	Open-text response	Link to review article:<br>https://www.sciencedirect.com/science/articl

already been brought up by Klamath County Public Health:<br><br>1. Prenatal birth<br>2. Low birthweight<br>3. Small for gestational age<br><br>Additional outcomes that have been investigated in relationship to wildfire smoke exposure include:<br><br>• Pregnancy loss (stillborn, spontaneous abortion)<br>• Difficulty conceiving<br>• Respiratory function (asthma, COPD, Cough, wheeze, chest tightness, colds, rhinitis)<br>• Cognitive function<br>• Inflammation (white blood cell count, serum levels, etc.)<br><br>Please use the space below to list other outcomes that may be useful.		e/abs/pii/S1382668917302478
10. Please use this space to provide any thoughts you may have about this project, your involvement, or other people you think we should reach out to.	Open-text response	n/a
11. Thank you for your input! If you have any additional questions, please direct them to (contact email).. We look forward to continuing to work with you.	n/a	n/a

Supplemental Table S3. Health Outcomes Survey

**Question**	**Response Options**	**Embedded Information**
**BLOCK 1:** Introductory text		
1. At our last meeting, we focused on the chemicals of interest and potential data collection tools.<br><br>For our meeting on December 7th, we will focus on potential health outcomes in addition to preterm birth and low birthweight.<br><br>As a reminder, our meeting notes, recordings and other information are available on this webpage.	**n/a**	**n/a**
2. To make the best use of our time, we are using this survey to gather information from you regarding the types of health outcomes we should collect and how we should collect them.	**n/a**	**n/a**

Your answers to these questions help us learn more about your interests. We will continue to discuss all of this at the next meeting. We do not expect to make any decisions at this point, but will continue to use your input as we go through this process.		
Block 2: Health Outcomes		
3. Here, we have listed health outcomes associated with wildfire smoke that can be measured prior to, or during, pregnancy. Please move the marker to show which you think are the most important to collect, or you have the most interest in collecting.<br><br>• Difficulty conceiving<br>• Miscarriage<br>• Stillbirth/ fetal death<br>• Other<br><br>Not sure what you might be interested in? You can mark that in the question below as well.	Slider<br>0 = no interest, 5 = high interest<br>Not sure<br>Other (open-text response)	**n/a**
4. Listed below are infant health outcomes that have been associated with wildfire smoke. Please move the marker to show which you think are the most important to collect, or you have the most interest in collecting.<br><br>• Preterm birth<br>• Gestational age at birth<br>• Low birth weight<br>• Small for gestational age<br>• NICU admissions<br>• Other	Slider<br>0 = no interest, 5 = high interest<br>Not sure<br>Other (open-text response)	**n/a**
5. Finally, there are health outcomes associated with the child as it grows, from infant, to toddler, to child. Please move the marker to show which you think are the most important to collect, or you have the most interest in collecting.<br><br>• Infant mortality<br>• Immune dysregulation<br>• Respiratory health<br>• Developmental milestones<br>• Cognitive outcomes (ADHD, etc.)	Slider<br>0 = no interest, 5 = high interest<br>Not sure<br>Other (open-text response)	**n/a**

• Other		
6. The following tools or samples can be used to collect health information. (Many of these can also be used to assess chemical exposure). Rank how important each one is to you. In your mind, how important do you think each data type would be for this study?<br><br>• Blood Samples (can be used to measure immune status)<br>• Placenta<br>• Vital statistics<br>• Surveys/ Questionnaires<br>• Health data (medial records, lung function, etc.)<br>• Developmental Scales<br>• Other<br><br>Not sure what you might be important? You can mark that in the question below as well.	Slider<br>0 = not important, 5 = very important<br>Not sure<br>Other (open-text response)	Links to online resources embedded:<br><br>**Vital statistics:** www.cdc.gov/nchs/about/factsheets/factsheet_nvss_improvements.htm<br><br>**Developmental Scales:** www.ncbi.nlm.nih.gov/books/NBK567715/
7. Much of our discussions have focused specifically on infant health. How interested are you in a longitudinal study, where children would be followed for several years after birth, to look at outcomes like respiratory health and developmental health?<br><br>Please rate your level of interest in a longitudinal study	Slider<br>0=not interested, 5=very interested, Not sure	n/a
8. Please use this space to provide any thoughts you may have about this project, your involvement, or other people you think we should reach out to.	Open-text response	n/a
9. If there are any papers or presentations you think we should be aware of, you may upload them here or email them to (contact email).. For URLs, please enter them in the space above.		Box provided to upload documents/paste urls

Supplemental Table S4. Results of consensus building using a combination of surveys and online discussions

	Selected	Rationale

$PM_{2.5}$	Yes	Highly ranked by team members; Previously associated with adverse outcomes in perinatal health; Easy to measure with low-cost monitors.
PAHs	Yes	Highly ranked by team members; Known pollutant in wildfire smoke.
Semi-volatile and volatile organic compounds (SVOCs & VOCs)	Yes	A team member had developed a screen for over 1500 chemicals (Bergmann et al. 2018) that included endocrine disruptors, flame retardants, and more. Team members found these chemicals interesting, and thus were in consensus of including them if an analytic method existed.
Metals	No	No interest from either researchers or community partners.
CO	Yes	Added as a pollutant of interest during the meeting by researchers.
**Collection Methodology & Tools**	The CCART identified multiple data collection methodologies. Within each methodology, there are multiple types of tools; the team met to first identify methodologies, and then to identify specific tools.	
	**Selected**	**Rationale**
Direct sampling (e.g., wristband, air monitor, hand wipes)	Yes	Given the research question, the team quickly came to consensus that personal sampling should be used.<br>**$PM_{2.5}$** - This type of sampler was originally selected. The instrument chosen (filter-based active PM2.5 personal sampler) had a steep learning curve and would require each participant to download an app and program the device to start and stop. Community partners later opted to remove the personal $PM_{2.5}$ sampler and replace it with a residential monitor (DylosPro).<br>**PAHs and other SVOCs/VOCs** - KCPHs and TOTs were initially interested in silicone wristbands. Team members had a broad interest in pollutants of interest (see above) which could be measured by the wristband.
Biological sampling (blood, urine, placenta)	No	One team member ranked each biological sample as essential. However, during group discussion many individuals familiar with the community discouraged the use of blood and urine, as many of their patients had listed concerns that such samples would be drug-tested, and therefore often chose not to participate in studies with this type of data collection.
Indirect samples – residential air	Yes	The team opted to place a residential $PM_{2.5}$ monitor and CO monitor in the home of each participant.
Indirect samples – ambient air	Yes	There are two air monitoring stations in Klamath County, maintained by KCPH. The team was in agreement that existing monitoring data should be supplemented with additional environmental samples for filter-based $PM_{2.5}$ and CO, as well as environmental passive samplers for PAHs and SVOCs/VOCs.
Survey(s)	Yes	The team discussed survey fatigue and a sensitivity around not burdening pregnant individuals. However, without EHR or other data, it was essential to use surveys for several areas of the study.<br><br>Team members shared three validated surveys that could be used with pregnant individuals to screen for low medical risk pregnant individuals and to ensure we understood any pertinent health history when interpreting results.<br><br>Community partners recommended that a daily respiratory survey during the sampling periods be very short (< 2 minutes) and

		delivered by text based on their experiences and response rates within their programs.
Existing data (e.g., Vital Statistics, Electronic Health Records)	No	Given challenges in accessing individual data across multiple hospitals/healthcare facilities, plus time constraints of the project, the team opted against electronic health records
**Health Outcomes**	Health outcomes were identified based on a review of the literature assessing wildfire smoke exposure during pregnancy. Health outcomes from conception to birth were assessed by the team.	
	**Selected**	**Rationale**
Difficulty conceiving	No	Community partners including KCPH and TOTs requested a focus on adverse birth outcomes.
Miscarriage/Stillbirth	---	The study did not explicitly focus on these outcomes, but participants were asked to note if their pregnancy resulted in a live birth.
Low birth weight	Yes	This information was collected via survey at the conclusion of the study.
Preterm birth	Yes	
Gestational age at birth	Yes	
Small for gestational age	Yes	
NICU admissions & Apgar score	Yes	
Respiratory health – parent & child	Yes	While the possible health outcomes initially identified did not include respiratory outcomes, the team identified this outcome and agreed it should be collected via self-reported survey responses.
Child development	No	There was interest within team members on a longitudinal study design that could focus on developmental milestones and cognitive outcomes, but that was deemed outside the scope of this pilot project.
**Study Design**	The team was asked for input on several aspects of the design, to include eligibility criteria and repeat measures.	
Repeat measures	The study was designed to sample pregnant individuals before the start of wildfires in Klamath, and then again during a period of wildfire smoke inundation.	
Eligibility criteria	Agreed on low-risk pregnancy criteria. Less than 16 weeks, singleton, 16-40 years of age, low medical risk, no or well controlled asthma, non-smoking.	
Feasibility	Given concerns across the team about overburdening pregnant individuals, feedback from participants on the level of difficulty of this study (e.g., instructions, setup, daily activities) was important to inform future studies.	

Supplemental Table S5. Surveys adapted or developed for the pilot project

**Survey Tool**	**Description**	**Citation**
Consent & Enrollment Survey	10 questions (consent)<br>88 questions (enrollment)<br>Enrollment survey includes demographic info, pregnancy history, exposure	Based on prior work conducted by the research team, and established surveys:<br>1. The British Medical Research Council Respiratory Questionnaire (validated)<br>2. American Thoracic Society and National Heart & Lung Institute – Division of Lung Disease Respiratory Questionnaire (ATS-DLD-78-A) (validated)

	history, COVID19 exposures, respiratory health, cancer history, smoke exposures, communications	3. 2020 BRFSS Questionnaire. Centers for Disease Control and Prevention. (validated) https://www.cdc.gov/brfss/questionnaires/pdf-ques/2020-BRFSS-Questionnaire-508.pdf<br>4. Environmental influences on Child Health Outcomes (ECHO) COVID-19 Questionnaire – Adult Primary Version. April 9, 2020.<br>5. Klamath County Public Health New Baby Questionnaire<br>6. 2015 Bastrop County CASPER Questionnaire (Kirsch, K.R., Feldt, B.A., Zane, D.F., Haywood, T., Jones, R.W. and Horney, J.A., 2016. Longitudinal community assessment for public health emergency response to wildfire, Bastrop County, Texas. Health security, 14(2), pp.93-104.)<br>7. American College of Medical Genetics. 1999. Sample Cancer Family History Questionnaire from Genetic Susceptibility to Breast and Ovarian Cancer: Assessment, Counseling and Testing Guidelines. https://www.ncbi.nlm.nih.gov/books/NBK56946/
Daily health survey	2 questions<br>Study participants are asked whether they see or smell wildfire smoke & to indicate their symptoms<br><br>Delivered via daily text for six days during the sampling period	These questions were based off a prior study: Rohlman, D., Dixon, H.M., Kincl, L., Larkin, A., Evoy, R., Barton, M., Phillips, A., Peterson, E., Scaffidi, C., Herbstman, J.B. and Waters, K.M., 2019. Development of an environmental health tool linking chemical exposures, physical location and lung function. *BMC public health*, *19*, pp.1-14.
Birth Outcome Survey	10 questions<br>Study participants are asked about birth outcomes, including stage of pregnancy, sex, weight, complications, and Apgar score.<br><br>Delivered 3 months after the second sampling period	These questions were based off an unpublished survey from the Bio-Specimen Assessment of Fire Effects Study (B-SAFE) study and was adapted by the CCART: Schmidt, R (2022). Maternal Labor & Delivery and Newborn Questionnaire. University of California Davis. https://tools.niehs.nih.gov/dr2/index.cfm/resource/24276
Feasibility Survey	14 questions<br>Study participants are asked about their experience in the study including overall difficulty, clarity of instructions, experience with	These questions were based off a prior study and adapted by the CCART: Rohlman, D., Dixon, H.M., Kincl, L., Larkin, A., Evoy, R., Barton, M., Phillips, A., Peterson, E., Scaffidi, C., Herbstman, J.B. and Waters, K.M., 2019. Development of an environmental health tool linking chemical exposures, physical location and lung function. *BMC public health*, *19*, pp.1-14.

	wristbands and monitors, how to improve the study<br><br>Delivered twice after monitors and wristbands were returned.	
